# microRNAs and Markers of Neutrophil Activation as Predictors of Early Incidental Post-Surgical Pulmonary Embolism in Patients with Intracranial Tumors

**DOI:** 10.3390/cancers12061536

**Published:** 2020-06-11

**Authors:** Julia Oto, Emma Plana, María José Solmoirago, Álvaro Fernández-Pardo, David Hervás, Fernando Cana, Francisco España, Andrea Artoni, Paolo Bucciarelli, Giorgio Carrabba, Silvia Navarro, Giuliana Merati, Pilar Medina

**Affiliations:** 1 Haemostasis, Thrombosis, Atherosclerosis and Vascular Biology Research Group, Medical Research Institute Hospital La Fe (IIS La Fe), 46026 Valencia, Spain; juliaotomartinez@gmail.com (J.O.); plana_emm@gva.es (E.P.); sol_mjo@gva.es (M.J.S.); alvarofernandezpardo@gmail.com (Á.F.-P.); fernando_cana@iislafe.es (F.C.); espanya_fra@gva.es (F.E.); navarro_silros@gva.es (S.N.); 2 Angiology and Vascular Surgery Service, La Fe University and Polytechnic Hospital, 46026 Valencia, Spain; 3 Data Science, Biostatistics and Bioinformatics Unit, Medical Research Institute Hospital La Fe (IIS La Fe), 46026 Valencia, Spain; bioestadistica@iislafe.es; 4 A. Bianchi Bonomi Hemophilia and Thrombosis Centre, Fondazione IRCCS Ca’Granda Ospedale Maggiore Policlinico, 20122 Milan, Italy; andrea.artoni@policlinico.mi.it (A.A.); paolo.bucciarelli@policlinico.mi.it (P.B.); giuliana.merati@unimi.it (G.M.); 5 Neurosurgery Unit, Fondazione IRCCS Ca’ Granda Ospedale Maggiore Policlinico, 20122 Milan, Italy; giorgio.carrabba@policlinico.mi.it

**Keywords:** microRNA, neutrophil activation, pulmonary embolism, glioma, meningioma, cancer, venous thromboembolism

## Abstract

Venous thromboembolism (VTE) is a common complication of cancer that severely increases morbidity and mortality. Patients with intracranial tumors are more likely to develop VTE than patients with cancers at other sites. Conversely, limited tools exist to identify patients with high thrombotic risk. Upon activation, neutrophils release their content through different mechanisms triggering thrombosis. We explored the ability of microRNAs (miRNAs) and plasma markers of neutrophil activation measured before surgery to predict the risk of early post-surgical pulmonary embolism (PE) in glioma and meningioma patients. We recruited and prospectively followed 50 patients with glioma and 50 with meningioma, 34% of whom in each group developed an early objectively-diagnosed post-surgical PE. We measured miRNA expression and neutrophil markers (cell-free DNA, nucleosomes, calprotectin and myeloperoxidase) before surgery. In glioma patients, we adjusted and validated a predictive model for post-surgical PE with 6 miRNAs: miR-363-3p, miR-93-3p, miR-22-5p, miR-451a, miR-222-3p and miR-140-3p (AUC = 0.78; 95% Confidence Interval (CI) [0.63, 0.94]) and another with cfDNA and myeloperoxidase as predictors (AUC = 0.71; 95% CI [0.52, 0.90]). Furthermore, we combined both types of markers and obtained a model with myeloperoxidase and miR-140-3p as predictors (AUC = 0.79; 95% CI [0.64, 0.94]). In meningioma patients we fitted and validated a predictive model with 6 miRNAs: miR-29a-3p, miR-660-5p, miR-331-3p, miR-126-5p, miR-23a-3p and miR-23b-3p (AUC = 0.69; 95% CI [0.52, 0.87]). All our models outperformed the Khorana score. This is the first study that analyzes the capability of plasma miRNAs and neutrophil activation markers to predict early post-surgical PE in glioma and meningioma patients. The estimation of the thrombotic risk before surgery may promote a tailored thromboprophylaxis in a selected group of high-risk patients, in order to minimize the incidence of PE and avoid bleedings.

## 1. Introduction

Cancer patients have a higher risk of venous thromboembolism (VTE) than non-cancer patients. As a consequence, the prognosis of cancer patients is worsened, with an increase in morbidity and mortality that exacerbates health costs [1]. Patients with intracranial tumors are more likely to develop VTE than patients who have cancers at other sites [2]. Accordingly, high-grade brain tumors correlate with a higher rate of VTE, including both deep vein thrombosis and pulmonary embolism (PE), being glioma one of the most thrombogenic among intracranial tumors [2]. The frequency of VTE after brain surgery is further increased [3]. However, it is different in malignant brain tumors such as glioma than in benign tumors such as meningioma, where operated patients have a risk of VTE up to 26% [4] and 30% [5,6], respectively. The underlying mechanisms responsible for the increased VTE risk seem to be heterogeneous, including factors related to the tumor itself, to the patient, to other physiopathological mechanisms (e.g., inflammation) as well as iatrogenic factors [7,8]. Tumor cells seem to induce hypercoagulability through multiple mechanisms, such as the production of procoagulant and proaggregating molecules (e.g., tissue factor), and the release of pro-inflammatory cytokines that activate endothelial cells, platelets and leukocytes. A direct effect of haemostasis in enhancing angiogenesis, cell survival and metastasis has also been observed [9,10].

Because VTE is a frequent complication of cancer that strongly increases morbidity and mortality in these patients, novel biomarkers are needed to identify cancer patients with high VTE risk. Furthermore, the postoperative diagnosis of VTE in neurosurgical patients is of paramount importance provided that the use of anticoagulant therapy increases the risk of intracranial hemorrhagic complications [5].

microRNAs (miRNAs) are small non-coding RNAs that regulate protein expression, and have been suggested as regulatory molecules and biomarkers in practically all cancer types [11]. In the particular scenario of intracranial tumors, several miRNAs have been proposed as biomarkers in glioma [12,13,14,15,16] and meningioma [17,18,19], and even these molecules have been proposed to have therapeutic benefits in these tumors [20,21]. However, to date, the role of miRNAs in the stratification of VTE risk in patients with intracranial tumors has never been explored.

Once activated, neutrophils a crucial role in host defense by phagocytosis, degranulation and also by neutrophil extracellular trap (NET) formation. NETs are extracellular networks of DNA, histones and proteins (calprotectin, myeloperoxidase, elastase, etc.) released by neutrophils in response to an inflammatory stimulus [22] or to the presence of pathogens, in a process called NETosis [22]. Moreover, NETs prompt coagulation [23], thus inducing cancer-associated thrombosis, since neutrophils activated by cancer cells produce more NETs than those activated by other mechanisms [24]. The neutrophil-lymphocyte ratio has been widely explored as predictor for overall survival in intracranial tumor patients [25,26], however no previous studies have addressed the role of neutrophil activation markers in these tumors or their thrombotic complications.

Our main goal was to evaluate the ability of miRNAs and neutrophil activation markers, measured before surgery, to predict the risk of early post-surgical PE in glioma and meningioma patients, in order to identify a subgroup of patients at a higher risk of PE who may deserve a tailored thromboprophylaxis.

## 2. Results

### 2.1. Clinical Characteristics of the Study Subjects

In the original cohort, 59 consecutive patients with glioma and 93 consecutive patients with meningioma were recruited and prospectively followed. In 18 patients with glioma (31%) and in 36 with meningioma (39%) an early post-surgical incidental PE was objectively diagnosed. From the original cohort, we selected for this study 50 patients from each tumor type, 34% of which (17 patients for each tumor type) had developed an early post-surgical PE. These patients were all the available glioma patients with follow-up completed at the time of the validation stage commencement, and a random selection of meningioma patients with an incidence of PE similar to that of the whole original cohort. The clinical characteristics of the study subjects studied herein are depicted in Table 1 and raw data from each individual can be consulted in Table S1.

No differences in age were observed between both clinical groups. Female sex was more frequent in meningioma than in glioma patients (*p* = 0.0439), consistent with the higher prevalence of this tumor type in women. All patients had negative baseline lung perfusion scans, thus indicating that none had pre-surgical asymptomatic PE. Those patients who developed an early post-surgical PE diagnosed with perfusion lung scan and confirmed with CT scan, underwent a color-Doppler compression ultrasound of the lower limbs to check for deep vein thrombosis. None of them had a positive result, thus discarding the presence of deep vein thrombosis. An intracranial hemorrhagic complication occurred in three patients with glioma (6%; two during surgery and one after surgery), and in two patients with meningioma (4%, both during surgery). Thirty-four of the 50 patients with glioma (68%) and 30 of the 50 patients with meningioma (60%) had one or more comorbidities, mostly cardiovascular. These selected patients were representative of the whole sample set in terms of comorbidities. In accordance with meningioma being a benign tumor and glioma a malignant tumor, a higher proportion of grade III and IV tumors were observed in glioma patients and a higher proportion of grade I and II tumors were observed in meningioma patients. Additionally, a higher proportion of patients with score 1 of Khorana were found in the glioma group (46%) compared to meningioma (20%), while most meningioma patients (80%) had a Khorana score of 0 (*p* = 0.0099). No overrepresentation of score 1 of Khorana occurred among PE patients.

Regarding tumor location, 12 gliomas (24%) were superficial and 38 were deep-seated (76%), while 15 meningiomas (30%) were located in the base of the skull and 35 (70%) were located in the cerebral convexity or falx. To rule out the possibility that the surgery performed had an impact on the occurrence of post-surgical PE, we compared the tumor size and the duration of the surgery (Table 1). Glioma tumors were bigger than meningiomas (24 and 16 cm^3^, respectively; *p* = 0.0481), while the duration of the surgery was similar between both tumor types (240 and 215 min, respectively; *p* = 0.606).

Regarding other clinical variables registered, significant differences were observed between glioma and meningioma patients in hemoglobin levels (median 13.7 and 12.9 g/dL, respectively; *p* = 0.0131), white blood cells (median 10.04 and 6.10 × 10^3^/mmc, respectively; *p* < 0.0001), neutrophil counts (median 6.03 and 3.66 × 10^3^/mmc, respectively; *p* < 0.0001) and activated partial thromboplastin time (APTT) ratio (median 0.81 and 0.94, respectively; *p* < 0.0001).

### 2.2. miRNA Expression Levels and Risk of Incidental Post-Surgical PE

At the initial screening stage, we studied before surgery the expression level of 179 miRNAs frequently present in human plasma in 10 glioma and 10 meningioma patients, five with post-surgical PE in each group (cases and controls) by using predefined panels. Of the 179 miRNAs comprised in the panel, 130 showed high quality signals (Ct < 36) in at least one study group (Table S1). Concerning the RNA controls used for RNA isolation (spike-in 2), cDNA synthesis (spike-in 6), and qPCR performance (spike-in 3), no differences were observed in any comparison made (Figure S1 and Table S1).

Regarding the best normalizer for the expression level of each miRNA studied, the Comprehensive Ranking of RefFinder rendered miR-93-5p as the one with the highest stability and the lowest biological variance among all glioma and meningioma samples (Figure S2A and Table S1). Additionally, miR-93-5p was the candidate normalizer with the best expression value (lowest Ct) (median Ct = 29.17 [28.48–30.10]), compared to miR-423-5p (median Ct = 32.85 [32.32–33.99]), miR-425-5p (median Ct = 31.06 [30.30–31.54]), miR-191-5p (median Ct = 32.10 [31.35–33.03]) and miR-103a-3p (median Ct = 30.18 [29.56–31.49]) (Figure S2B). Thus, we normalized the expression level of each miRNA studied by that of miR-93-5p using the 2^-ΔΔCT^ method. Next, we validated the predictive models obtained in the independent cohort of 40 glioma and 40 meningioma patients.

#### 2.2.1. Glioma

**Figure 1:**
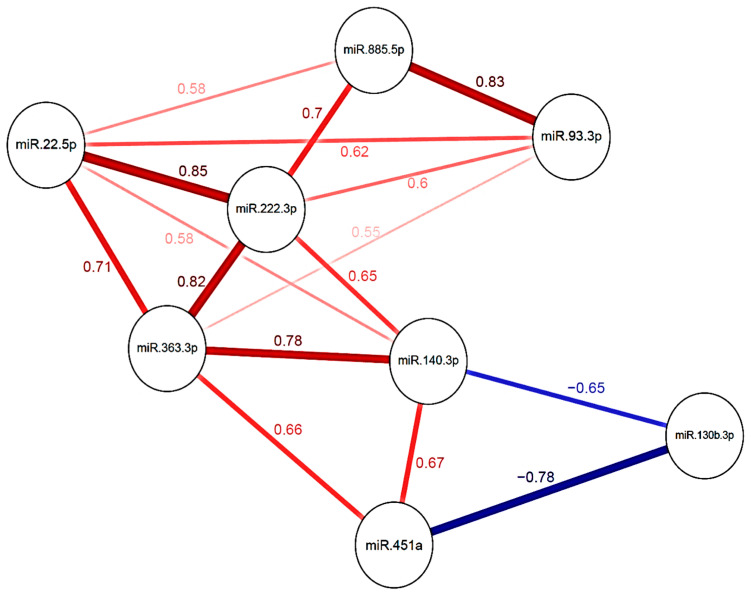
Correlation among the miRNAs included in the predictive model of post-surgical PE in glioma patients. Spearman correlation coefficients between two miRNAs are depicted next to the lines. Positive correlations are represented by red positive correlation values and red lines; negative correlations are represented by blue negative correlation values and blue lines. The degree of correlation is represented by the intensity of the number and the line and by the thickness of the lines, the more intense and thicker the line between two miRNAs, the strongest the correlation.

In the screening stage, we adjusted a multivariable elastic net logistic regression model for PE risk with the miRNA expression levels before surgery. The model included eight miRNAs as predictors: miR-363-3p, miR-93-3p, miR-22-5p, miR-130b-3p, miR-885-5p, miR-451a, miR-222-3p and miR-140-3p. The effect of each miRNA was stated as standardized odds ratio (OR), reporting an increase of one standard deviation in the miRNA expression level. Accordingly, these miRNAs had standardized ORs ranging from 0.77 to 1.1 (Table 2). With this predictive model of PE we obtained an optimism-corrected area under the curve (AUC) of 0.78 (*p* = 0.003). Importantly, there was a strong correlation among the different miRNAs included in our predictive model (Figure 1).

**Figure 2:**
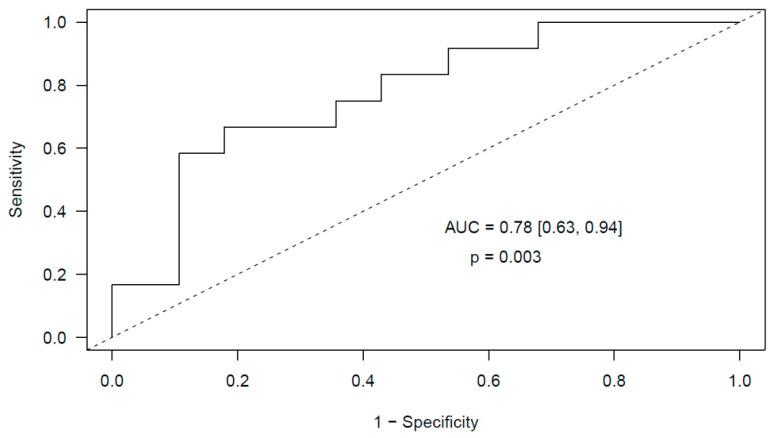
Validated ROC curve obtained from the multivariable elastic net logistic regression predictive model that contains six miRNAs measured before surgery (miR-363-3p, miR-93-3p, miR-22-5p, miR-451a, miR-222-3p and miR-140-3p) as predictors of post-surgical PE in glioma patients.

Next, we validated the predictive ability of our model in the cohort of 40 glioma patients. In them, we could quantify by real-time quantitative reverse transcription PCR (RT-qPCR) using specific primers the expression level of six of the eight miRNAs included in the model: miR-363-3p, miR-93-3p, miR-22-5p, miR-451a, miR-222-3p and miR-140-3p. The remaining two miRNAs had a very low expression level thus not achieving an appropriate qPCR criteria (Ct < 35 and standard deviation between duplicates < 0.5) (Table S1). At this validation stage we obtained a receiver operator characteristic (ROC) curve AUC of 0.78 (95% Confidence Interval (CI) [0.63, 0.94], *p* = 0.003) (Figure 2).

Regarding the clinical variables studied, the APTT ratio was the only variable significantly increased in glioma patients who developed PE (*p* = 0.0139) (Table S2). Additionally, a higher proportion of grade IV tumors occurred in glioma patients who developed PE (*p* = 0.0183). However, the inclusion of the clinical variables registered did not improve the predictive model obtained. The formula for estimating the risk of post-surgical PE in glioma patients with this model (Equation (1)) was built with the coefficients provided by the model for each predictive variable, and is as follows:

(1) $$\mathrm{Pr}\left(PE\right)=\frac{e^{LP}}{1+e^{LP}}$$

where LP is given by the expression LP = −1.25 + 0.70 × miR-363-3p − 0.89 × miR-93-3p + 0.58 × miR-22-5p − 1.02 × miR-451a − 0.39 × miR-222-3p + 1.66 × miR-140-3p.

The sensitivity and specificity of our model can be calculated for different thresholds from a plot (Figure S3). Furthermore, applying the abovementioned formula of our predictive model, we estimated the thrombotic risk of each glioma patient before surgery. The median thrombotic risk before surgery of those patients who suffered a post-surgical PE was 0.46, while it was only 0.2 in the group of patients who did not suffer post-surgical PE.

Next, we ascertained the validated and predicted target proteins related to VTE of these miRNAs in the database miRWalk 2.0. Next, we combined these targets with the complement and coagulation cascades pathway from the Kyoto Encyclopedia of Genes and Genomes (KEGG) (Table 3).

#### 2.2.2. Meningioma

In the screening stage, we adjusted a multivariable elastic net logistic regression model for post-surgical PE with miR-660-5p as predictor. Furthermore, with the Random Forest regression test we obtained a predictive model of post-surgical PE with six miRNAs: miR-29a-3p, miR-660-5p, miR-331-3p, miR-126-5p, miR-23a-3p and miR-23b-3p (Table 4 and Figure S4). These miRNAs had a fold-change ranging from −1.59 to 2.20.

**Figure 3:**
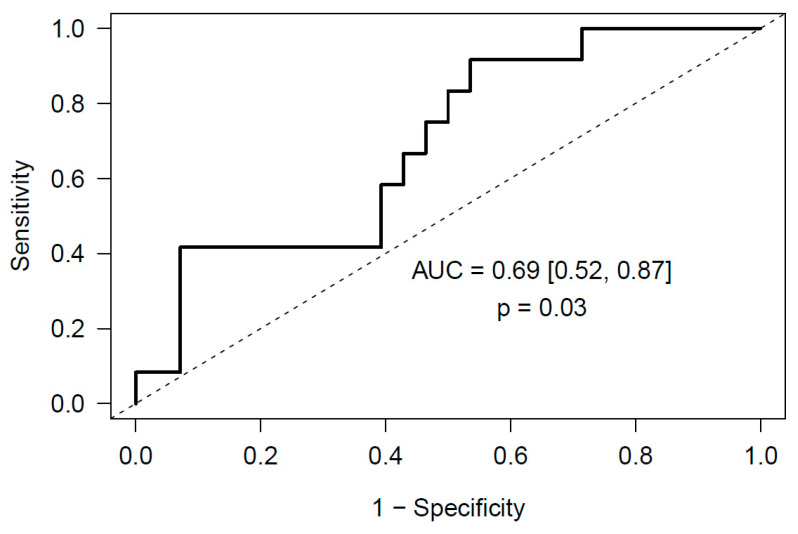
Validated ROC curve obtained from the Random Forest regression predictive model that includes 6 miRNAs measured before surgery (miR-29a-3p, miR-660-5p, miR-331-3p, miR-126-5p, miR-23a-3p, miR-23b-3p) as predictors of post-surgical PE in meningioma patients.

Next, we validated the predictive ability of our model in the cohort of 40 meningioma patients. In them, we could quantify by RT-qPCR using specific primers the expression level of all 6 miRNAs comprised in the model. In this validation stage, we obtained a ROC curve AUC of 0.69 (95% CI [0.52, 0.87], *p* = 0.03) (Figure 3).

Regarding the clinical variables studied, the duration of surgery and platelet count were significantly increased in those meningioma patients who developed PE (*p* = 0.00258 and *p* = 0.0029, respectively). Similarly, a higher proportion of comorbidities catalogued as miscellanea (previous surgeries, other neoplasms, psychiatric disorders) occurred in meningioma patients with PE (*p* = 0.0400). However, the inclusion of the clinical variables registered did not improve the predictive model obtained.

The formula for estimating the risk of post-surgical PE in meningioma patients with this model (Equation (2)) was built with the coefficients provided by the model for each predictive variable, and is as follows:

(2) $$\mathrm{Pr}\left(PE\right)=\frac{e^{LP}}{1+e^{LP}}$$

where LP is given by LP = −1.32 − 0.90 × miR-660-5p + 0.88 × miR-23b-3p − 1.3 × miR-23a-3p − 0.03 × miR-29a-3p + 1.27 × miR-331-3p − 1.57 × miR-126-5p.

Subsequently, we ascertained the validated and predicted target proteins related to VTE of these miRNAs in the database miRWalk 2.0. Finally, we combined these targets with the complement and coagulation cascades pathway from KEGG (Table 5).

### 2.3. Markers of Neutrophil Activation and Risk of Incidental Post-Surgical PE

#### 2.3.1. Glioma

To explore the ability of neutrophil activation markers, measured before surgery, to predict the risk of early post-surgical PE in our patients, we quantified cfDNA, nucleosomes, MPO and calprotectin using specific assays and we compared their levels with the Wilcoxon-Mann-Whitney test between those patients who suffered post-surgical PE and those who did not (cases and controls). The neutrophil counts before surgery were similar in the two groups (median 6.52 and 5.6 × 10^3^/mmc, respectively; *p* = 0.1345) (Table S2). The values of these markers obtained for each individual studied can be consulted in Table S1. We found an increase in myeloperoxidase (MPO) levels in patients who suffered PE (118.5 ng/mL) compared to those who did not (75.7 ng/mL) (*p* = 0.028) as occurred with calprotectin levels (2051 vs. 1368 ng/mL, respectively; *p* = 0.06). We did not observe considerable differences in plasma cell-free DNA (cfDNA) levels (1180 vs. 1146 ng/mL, respectively; *p* = 0.31) or nucleosomes levels (0.35 vs. 0.22 U, respectively; *p* = 0.23). We found a significant correlation between cfDNA and MPO levels (Spearman r = 0.323, *p* = 0.022), suggesting that neutrophils may be the source of these makers in plasma of glioma patients.

**Figure 4:**
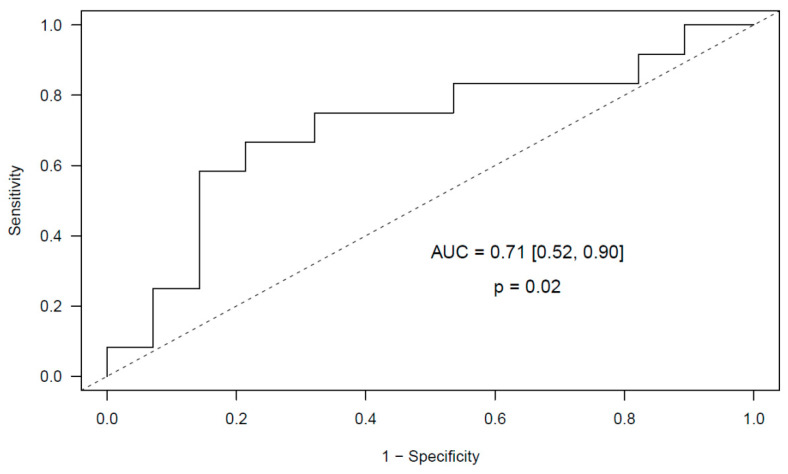
Validated ROC curve obtained from the multivariable elastic net logistic regression predictive model that includes MPO and cfDNA measured before surgery as predictors of post-surgical PE in glioma patients.

At the screening stage (10 glioma patients) we attained a reliable multivariable elastic net logistic regression model of post-surgical PE with cfDNA and MPO as predictors (AUC = 0.88, 95% CI [0.63, 1.00], *p* = 0.028). We validated this predictive model in the independent cohort of 40 glioma patients and obtained a validated AUC of 0.71 (95% CI [0.52, 0.90], *p* = 0.02) (Figure 4).

The inclusion of the clinical variables registered did not improve the predictive model obtained. The formula for estimating the risk of post-surgical PE in glioma patients with our model (Equation (3)) was built with the coefficients provided by the model for each predictive variable, and is the following:

(3) $$\mathrm{Pr}\left(PE\right)=\frac{e^{-1.96\times 0.021\times \mathrm{MPO}-0.001\times DNA}}{1+e^{-1.96\times 0.021\times \mathrm{MPO}-0.001\times DNA}}$$

**Figure 5:**
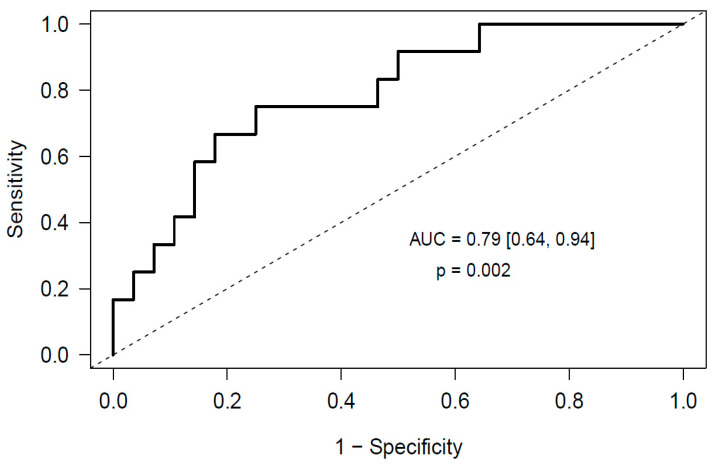
ROC curve obtained from the multivariable elastic net logistic regression predictive model that includes MPO and miR-140-3p measured before surgery as predictors of post-surgical PE in glioma patients.

To evaluate whether markers of neutrophil activation combined with miRNAs improved the predictive capability of the individual models, we adjusted a new multivariable elastic net logistic regression model that included MPO and miR-140-3p as predictors, achieving an AUC of 0.79 (95% CI [0.64, 0.94], *p* = 0.002) (Figure 5), which became 0.76 after correction for optimism.

Finally, we wanted to compare the performance of our predictive models with the Khorana score in our samples. The ROC curve of the Khorana score in our samples achieved an AUC of 0.52 (95% CI [0.31, 0.72], *p* = 0.72) (Figure S5). Therefore, our predictive models outperformed the Khorana score in the prediction of post-surgical PE in glioma patients.

#### 2.3.2. Meningioma

We compared the levels of the neutrophil activation markers studied between meningioma patients who suffered post-surgical PE and those who did not with the Wilcoxon-Mann-Whitney test, and we did not observe any difference in these parameters (data not shown). The neutrophil counts before surgery were similar in both groups (median 3.64 and 3.66 × 10^3^/mmc, respectively; *p* = 0.4203) (Table S3). The values of these markers obtained for each individual studied can be consulted in Table S1. Moreover, in meningioma patients the markers of neutrophil activation were not predictors of early incidental post-surgical PE.

## 3. Discussion

The occurrence of VTE in cancer patients increases morbidity, mortality, and medical expenses. The poor prognosis and the increase in disease burden related to VTE in cancer patients urges the need for the elaboration of risk stratification models to identify those patients at the highest risk to suffer VTE [27]. Despite the use of thromboprophylaxis in glioma and meningioma patients, a subgroup still develops post-surgical PE [2,3]. This clearly indicates that standard thromboprophylaxis is not adequate for all patients and those at a higher thrombotic risk are in need of a tailored dose and/or duration. Moreover, the bleeding risk of a standard thromboprophylaxis dose in low-risk patients reinforces the need to tailor thromboprophylaxis, especially in neurosurgical patients undergoing a craniotomy for brain tumor resection where the bleeding risk is approximately 3% [5]. For this purpose, biomarkers like D-dimer, sP-selectin and parameters of the thrombin generation test have been implemented in the existing risk stratification models that comprehend clinical variables such as cancer location, blood count parameters, and body mass index [1]. Indeed, efforts have been made to discover specific risk factors for thrombosis in patients with glioma and meningioma [28]. However, the degree of specificity of these models is low and cannot identify most of the cancer patients that will undergo a VTE during the progress of their disease. Although several clinical variables in our study increased with PE occurrence in glioma patients (APTT ratio and grade IV tumors) and meningioma patients (duration of surgery, platelet count and miscellanea comorbidities), none of the clinical variables registered were able to predict post-surgical PE in our patients. Thus, additional improvement of risk appraisal is needed to identify those cancer patients at high or low VTE risk to tailor thromboprophylaxis.

In our study we have firstly proved the utility of miRNAs and neutrophil activation markers as biomarkers for predicting early post-surgical PE in patients with intracranial tumors, namely glioma and meningioma. We have obtained a predictive model for early post-surgical PE in glioma patients that includes a profile of 6 miRNAs as predictors, identified in samples collected before surgery: miR-363-3p, miR-93-3p, miR-22-5p, miR-451a, miR-222-3p and miR-140-3p. This validated model achieves an AUC of 0.78 (95% CI [0.63, 0.94], *p* = 0.003). These miRNAs have been previously studied in intracranial tumors and in other malignancies. miR-363-3p has been related to lung adenocarcinoma [29] and glioblastoma [30]. miR-93-3p has been postulated as diagnostic biomarker for triple negative breast cancer [31] and renal cell carcinoma [32]. miR-22-5p seems implicated in tumorigenesis in breast cancer cells [33] and has been postulated to repress cancer progression by inducing cellular senescence [34]. miR-451a has been related to proliferation, invasion, apoptosis and stemness in glioma [35] and other types of cancer, like renal cell carcinoma [36] and colorectal [37]. Several reports have implicated miR-222 in glioma [38,39] and miR-140 has also been reported in intracranial tumors [40].

In meningioma, we have obtained a predictive model for post-surgical PE that includes a profile of 6 miRNAs as predictors: miR-29a-3p, miR-660-5p, miR-331-3p, miR-126-5p, miR-23a-3p, miR-23b-3p. This validated model achieves an AUC of 0.69 (95% CI [0.52, 0.87], *p* = 0.03). miR-29a-3p has been related to many different types of cancer, like gastric cancer [41] and hepatocellular carcinoma [42]. miR-660-5p seems to regulate the proliferation, migration, and invasion of human breast cancer cells [43] and it has been related to cell migration, invasion, proliferation and apoptosis in renal cell carcinoma [44]. miR-331-3p has been recently postulated as predictor of recurrence in esophageal adenocarcinoma [45] and as regulator in pancreatic cancer [46]. miR-126-5p has been studied in cervical cancer [47] and renal cell carcinoma [48]. miR-23a-3p and miR-23b-3p regulate the expression of proapoptotic proteins in human pancreatic beta-cells [49].

To delve into the biological mechanism(s) regulated by these miRNAs, we identified their targets and the pathways where they are involved. We found that most miRNAs are potential regulators of several components along the complement and coagulation cascades pathway. Interestingly, 12 targets may be regulated by at least two different miRNAs as for example miR-93-3p and miR-22-5p that may both regulate the expression of tissue factor pathway inhibitor. Indeed, the human TFPI-2 has been previously related with cell invasion in glioma [50]. Additionally, six targets are regulated by our miRNAs both in glioma and meningioma: CR2, KNG1, PROS1, F9, MBL2 and F11, while different miRNAs are dysregulated in each cancer type. Our results support the complexity in the regulation of human biological pathways exercised by miRNAs, given that one miRNA targets many mRNAs within the same pathway and each mRNA is targeted by several miRNAs to guarantee a fine-tuned global regulation. In addition, the targets regulated by one miRNA can have opposite functions, and the final outcome of the miRNA could then be either pro-thrombotic or anti-thrombotic. Additional in vitro studies in cell cultures and in vivo in animal models will prove the final regulation of each predicted target and will shed light on the degree of participation of each miRNA in the global regulatory mechanisms in glioma and meningioma patients. To the best of our knowledge, ours is the first study that addresses the predictive role of plasma miRNAs for PE in glioma and meningioma patients. The study of these miRNAs in paired cancer tissue specimens may unravel the origin of the dysregulation.

Regarding the role of neutrophil activation markers, we observed an increase in MPO and calprotectin levels in glioma patients who underwent post-surgical PE. cfDNA and nucleosomes levels also were slightly increased in these patients. Differences in neutrophil counts may be the source of variation in plasma activation markers between both groups, however no differences in neutrophil counts were observed between glioma patients who suffered post-surgical PE and those who did not. Although the origin of these neutrophil activation markers could be other than neutrophils, we observed a significant correlation between cfDNA and MPO levels, suggesting that neutrophils may be the source of these makers in plasma of glioma patients. Furthermore, we attained a predictive model for post-surgical PE in glioma that includes cfDNA and MPO as predictors. This validated model achieves an AUC of 0.71 (95% CI [0.52, 0.90], *p* = 0.02). On the contrary, neutrophil activation markers were not good predictors of thrombotic complications in meningioma patients. In a similar strategy as that conducted in the present study, several neutrophil activation markers like DNA and MPO have been associated to other prothrombotic states [51,52,53,54,55,56] however, to date, this is the first study in which the predictive role of neutrophil activation markers for PE in glioma and meningioma patients is addressed. To further evaluate whether markers of neutrophil activation combined with miRNAs improved the predictive ability of the individual models in glioma patients, we adjusted a new comprehensive elastic net model that included MPO and miR-140-3p as predictors, achieving an AUC of 0.79 (95% CI [0.64, 0.94], *p* = 0.002).

Presently, the most used risk stratification tool for thrombosis in cancer patients is the Khorana score [57]. However, its limitations reveal that current tools to predict and monitor the risk of VTE are inadequate [58,59]. The Khorana score might not be appropriate for brain tumors, as the original study only included a few patients with these types of cancers. However, since the Khorana score is one of the most popular predictive tools used in clinical practice, we decided to include this score in our study, in order to compare it with our novel predictive tools for the occurrence of post-surgical PE in patients with brain tumors. All our predictive models described herein outperformed the widely used Khorana score.

Strengths of this study are the validation of our findings in an independent cohort of patients prospectively followed, and the exhaustive evaluation of patients at inclusion and during follow-up. A limitation of our study could be the sample size studied. However, the incidence of early post-surgical PE was sufficiently high to guarantee a reliable prediction model. Indeed, consistent with the high frequency of PE events in glioma and meningioma patients, 31% of patients with glioma and 39% with meningioma in the original cohort developed a post-surgical PE. The 50 patients with glioma and 50 with meningioma, who formed the study sample, represent almost the entire cohort of patients with glioma and a random selection of patients with meningioma, with an incidence of PE as high as 34%, similar to that of the original cohort.

## 4. Materials and Methods

### 4.1. Study Subjects

Fifty-nine consecutive patients with glioma and 93 with meningioma, candidates to removal of the intracranial tumor (primary or relapse) were recruited and prospectively followed between 2012 and 2016 at the Fondazione IRCCS Ca’ Granda Ospedale Maggiore Policlinico of Milan (Italy). For the purpose of this study, 50 patients with glioma and 50 with meningioma were selected, who had similar demographic and clinical characteristics to those of the original cohort. This selection was conducted as follows: the 50 glioma patients studied herein represent all the available patients with follow-up completed at the time of the validation stage commencing (out of 59 glioma patients from the original cohort), while the 50 meningioma patients represent a random selection of patients with an incidence of PE similar to the whole original cohort. Cancer was objectively diagnosed with brain CT scan or magnetic resonance. The tumor histological classification was done according to the 2007 World Health Organization (WHO) Classification of brain tumors in grade I, II, III and IV [60], being grade I the less severe and IV the most severe.

PE was objectively diagnosed by means of a perfusion lung scan. To rule out the existence of pre-surgical asymptomatic PE, patients were scanned at the visit before surgery. To diagnose post-surgical PE, all patients underwent a second perfusion lung scan within 2–7 days after surgery. In case of a positive result, a chest CT scan was performed to confirm the diagnosis. Only PE diagnosed with perfusion lung scan and confirmed with CT scan were considered. A color-Doppler compression ultrasound of the lower limbs was performed only in patients with a PE diagnosis to check deep vein thrombosis.

Clinical and demographic data were collected from all patients. Pre-operative co-morbidities were categorized in cardiovascular, respiratory, metabolic (diabetes mellitus, hypercholesterolemia, obesity, hyper- or hypothyroidism, chronic liver or renal disease) and miscellanea (previous surgeries, other neoplasms and psychiatric disorders). Karnofsky Performance Status (KPS) was documented before surgery and at patient’s discharge. Post-operative risk factors comprised the presence of neurological aggravation and walking complications. As standard clinical practice, antithrombotic prophylaxis with low molecular weight heparin (enoxaparin) was daily administered at a single dose of 4000 IU subcutaneously without any adjustment for body weight or renal function (since neither overweight patients nor patients with renal disease were present) beginning 24 h after surgery or later when intracranial bleeding was revealed at the post-operative brain CT scan. All patients wore elastic stockings in the perioperative period and none were treated with chemotherapy during the duration of the present study since it was limited to the first week after surgery. When VTE occurred, anti-coagulation was initiated with low molecular weight heparin personalized to each patient’s clinico-radiological picture (mainly 4000 IU twice a day).

The exclusion criteria were: patient’s refusal to participate in the study, previous history of VTE, need for anticoagulant or antiplatelet therapy for other reasons, and known blood coagulation disorders that contraindicated the use of antithrombotic prophylaxis.

All participants provided written informed consent. The study was conducted in accordance with the Declaration of Helsinki and the protocol was approved by the Ethics Committee of the Fondazione IRCCS Ca’ Granda Ospedale Maggiore Policlinico of Milan.

### 4.2. Blood Collection

Blood was obtained from all patients before surgery, collected in Vacutainer tubes (BD Diagnostics, Franklin Lakes, NJ, USA) containing 0.109 M trisodium citrate and then centrifuged at 1500× *g* for 30 min at 4 °C. Plasma was stored in aliquots at −72 °C until used. The following blood tests were also performed: complete blood count, kidney and hepatic function, prothrombin time (PT), APTT, fibrinogen and D-dimer plasma levels.

### 4.3. miRNA Studies

#### 4.3.1. RNA Isolation

Total plasma RNA (including miRNAs) was isolated with the miRNeasy Mini Kit (Qiagen, Hiden, Germany) according to manufacturer’s instructions with slight optimizations reported by our group [61]. An RNA carrier (tRNA, Ambion, Bleiswijk, The Netherlands) was added during the isolation to increase the final yield. Several synthetic RNAs (Spike-in kit UniRT, Exiqon, Vedbaek, Denmark) were included in different steps of the whole procedure to track the RNA isolation efficiency, cDNA synthesis and inter-plate qPCR performance. The concentration and purity of the RNA was spectrophotometrically evaluated in a NanoDrop ND-1000 system (Thermo Fisher Scientific, Wilmington, DE, USA). RNA was stored at −80 °C until used. No plasma sample was haemolized, determined at the absorbance of haemoglobin (412 nm).

#### 4.3.2. Quantification of the Expression Level of miRNAs

The expression level of miRNAs was quantified by RT-qPCR in two stages:

##### Screening Stage

Based on the quality of the isolated RNA, 10 glioma and 10 meningioma patients were selected (five with and five without post-surgical PE in each clinical group, representative of the whole cohort, cases and controls) and the miRNAs expression level was studied before surgery. For that aim, we used the Universal cDNA Synthesis Kit II (Exiqon, Vedbaek, Denmark) and the Serum/Plasma Focus microRNA PCR Panel V4 (Exiqon, Vedbaek, Denmark) with the ExiLENT SYBR Green Master Mix (Exiqon, Vedbaek, Denmark) were used in a LightCycler 480 II (Roche, Mannheim, Germany), as previously reported [56]. To ensure that miRNA quantification was not influenced by technical and interpersonal variability, we included the following internal controls: we assessed the RNA isolation step by adding the synthetic spike-in 2 RNA in every RNA isolation, the retrotranscription efficiency by adding the spike-in 6 RNA in every cDNA synthesis reaction, and the qPCR performance by measuring the inter-plate calibrator spike-in 3 RNA included in triplicate in every panel and a negative control in every qPCR reaction.

The selection of the most stable normalizer among all samples was performed with the comprehensive tool RefFinder that comprehends the computational programs geNorm, Normfinder, BestKeeper and the comparative delta-Ct method (https://www.heartcure.com.au/for-researchers/). The candidate normalizers evaluated were those miRNAs proposed by the Serum/plasma Focus microRNA PCR Panel V4: miR-423-5p, miR-425-5p, miR-93-5p, miR-191-5p and miR-103a-3p. Once selected, the expression level of each miRNA was normalized by the 2^-ΔΔCT^ method.

Next, a multivariable logistic regression model was adjusted to predict post-surgical PE in glioma and meningioma patients according to miRNAs levels before surgery.

##### Validation Stage

Once we identified a miRNA profile potentially capable of predicting a post-surgical PE event in glioma and meningioma patients, their expression level was measured in an independent and larger cohort of patients (40 patients with glioma and 40 with meningioma; 12 cases of PE in each group) at inclusion in duplicate. For each miRNA, specific miRCURY LNA miRNA PCR Assay (Exiqon, Vedbaek, Denmark) were employed. Each miRNA was measured in duplicate and a standard deviation <0.5 was considered satisfactory.

#### 4.3.3. Identification of miRNAs’ Targets

Once we selected a miRNA profile capable of predicting a PE event in these patients, we ascertained their validated and predicted target proteins related to VTE in the databases miRWalk 2.0 (http://zmf.umm.uni-heidelberg.de/apps/zmf/mirwalk2/) that comprehends 12 miRNA-target prediction programs. Next, these targets were combined with the complement and coagulation cascades pathway from KEGG (https://www.genome.jp/kegg/).

### 4.4. Quantification of Neutrophil Activation Markers

Different markers of neutrophil activation were quantified in the plasma sample obtained before surgery, following the strategy addressed in previous studies [51,52,53,54,55,56,62] and following manufacturer´s instructions. These markers were: cfDNA (Quant-iT PicoGreen dsDNA kit, Life Technologies, Eugene, OR, USA) and nucleosomes (Cell Death Detection ELISA^PLUS^ kit, Roche, Mannheim, Germany) as markers of the neutrophil nuclear content released upon NETosis; calprotectin (Human Calprotectin ELISA kit, Hycult Biotech, Uden, The Netherlands) as marker of cytoplasmic content; and MPO (Human MPO ELISA kit, Abnova, Taoyuan, Taiwan) as marker of granules content, both released upon neutrophil activation by different mechanisms.

### 4.5. Statistical Analysis

All statistical analyses were conducted in R (version v3.5.1). Continuous variables were expressed as median and interquartile range, and categorical variables as count and percentage. In the screening stage (10 patients with glioma and 10 with meningioma; 5 cases of PE in each group), elastic net logistic regression models for PE risk were adjusted for each tumor type using the miRNA expression levels and the levels of neutrophil activation markers before surgery. The formulas to calculate the risk of PE in each individual patient were built with the coefficients provided by the model for each predictive variable. Additionally, a random forest analysis was performed in meningioma. The predictive ability of the models was evaluated by estimating the optimism-corrected AUC for the ROC analysis, using 1000 bootstrap replicates. Next, this AUC was verified in the validation stage (40 patients with glioma and 40 with meningioma; 12 cases of PE in each group). For all the estimates, 95% CIs were calculated. The association of neutrophil activation markers with PE was evaluated by comparing the levels of every marker in both clinical groups (with and without post-surgical PE, cases and controls), and for each tumor type, with the Wilcoxon-Mann-Whitney test. Results were considered statistically significant at *p* < 0.05. The 50 patients for each tumor type of the whole study group (10 for the screening stage and 40 for the validation stage) represented almost the entire cohort of the 59 patients with glioma and a random selection of the 93 patients with meningioma, with an incidence of PE similar to that of the original cohort.

## 5. Conclusions

In our study we reveal that plasma miRNAs and markers of neutrophil activation, measured before surgery, may be suitable predictors of early incidental post-surgical PE in patients with intracranial tumors, namely glioma and meningioma. A good prediction could be obtained with the expression level of 6 miRNAs in glioma (miR-363-3p, miR-93-3p, miR-22-5p, miR-451a, miR-222-3p and miR-140-3p) and in meningioma (miR-29a-3p, miR-660-5p, miR-331-3p, miR-126-5p, miR-23a-3p and miR-23b-3p) patients, respectively. Furthermore, a good prediction could also be obtained in glioma patients with markers of neutrophil activation, cfDNA and MPO, measured before surgery. Remarkably, we explored the predictive ability of both regulatory mechanisms in combination, and a good prediction could be obtained with MPO and miR-140-3p before surgery.

In recent years, targeted therapy to achieve a personalized medicine has become a crucial goal in medicine. Certainly, in patients with intracranial tumors an uncomplicated brain surgery is essential to prevent the occurrence of VTE influenced by a deterioration of neurological and/or clinical conditions [5]. Therefore, once confirmed in a large independent cohort of prospectively recruited patients, the plausible implementation of our risk stratification tools may provide physicians the possibility to tailor the thromboprophylaxis in the high-risk subgroup of patients during the perioperative period and promote a closer follow-up to minimize the incidence and morbidity of VTE.

## Figures and Tables

**Table 1:** Baseline clinical characteristics of the study subjects.

Clinical Characteristic	Glioma Patients (*n* = 50)	Meningioma Patients (*n* = 50)
PE events, *n* (%)	17 (34)	17 (34)
Age, y	61 (51–70)	64 (50–71)
Female sex, *n* (%)	22 (44)	33 (66)
BMI, kg/m^2^	24.7 (22.2–27.4)	25.9 (21.1–29.7)
Comorbidities, *n* (%) Cardiovascular Respiratory Metabolic * Miscellanea ^†^	20 (40) 1 (2) 8 (16) 5 (10)	18 (36) 2 (4) 5 (10) 5 (10)
Pre-operative KPS ≥ 80, *n* (%)	47 (94)	49 (98)
Post-operative KPS ≥ 80, *n* (%)	44 (88)	47 (94)
WHO classification, *n* (%) Grade I Grade II Grade III Grade IV	0 (--) 6 (12) 8 (16) 36 (72)	45 (90) 5 (10) 0 (--) 0 (--)
Tumor location, *n* (%) Skull base Cerebral convexity-falx Superficial Deep-seated	0 (--) 0 (--) 12 (24) 38 (76)	15 (30) 35 (70) 0 (--) 0 (--)
Tumor dimension, cm^3^	24 (12.3–50.2)	16 (5,8–35.2)
Duration of surgery, min	240 (210–240)	215 (176.3–277.5)
Khorana score, *n* (%) 0 1	27 (54) 23 (46)	40 (80) 10 (20)
Hemoglobin, g/dL	13.7 (12.7–14.9)	12.9 (11.9–13.3)
WBC, ×10^3^/mmc	10.04 (6.64–12.10)	6.10 (4.92–7.69)
Neutrophils, ×10^3^/mmc	6.03 (3.99–7.26)	3.66 (2.95–4.62)
Platelets, ×10^3^/mmc	226 (188–248)	216 (189–278)
PT ratio	0.99 (0.93–1.09)	1.02 (0.97–1.08)
APTT ratio	0.81 (0.73–0.89)	0.94 (0.86–1.01)
Fibrinogen, mg/dL	229 (196–279)	257 (222–304)
D-dimer, ng/mL	218 (125–565)	167 (100–210)
CRP, mg/L	0.07 (0.03–0.18)	0.11 (0.06–0.26)
eGFR, mL/min/1.73 m^3^	93.6 (79.8–113.9)	90.8 (78.3–107.6)

Continuous variables are displayed as median and interquartile range. Categorical variables are displayed as count and percentage. * Diabetes mellitus, hypercholesterolemia, obesity, hyper- or hypothyroidism, chronic liver or renal disease. ^†^ Previous surgeries, other neoplasms, psychiatric disorders. PE, pulmonary embolism; BMI, body mass index; KPS, Karnofsky Performance Status; WHO, World Health Organization Classification of brain tumors; WBC, white blood cells; PT, prothrombin time; APTT, activated partial thromboplastin time; CRP, C-reactive protein; eGFR, estimated glomerular filtration rate.

**Table 2:** miRNAs included in the multivariable elastic net logistic regression predictive model of post-surgical PE in glioma patients attained in the screening stage. miRNA sequences detailed in accordance with miRBase 22.1. Fold-change expresses the ratio of the average expression level of a miRNA in glioma patients who suffered a post-surgical PE event and those who did not.

miRNA	Sequence	Standardized OR	Fold-Change
miR-363-3p	aauugcacgguauccaucugua	0.85	−1.79
miR-93-3p	acugcugagcuagcacuucccg	0.91	−1.75
miR-22-5p	aguucuucaguggcaagcuuua	0.99	−2.50
miR-130b-3p	cagugcaaugaugaaagggcau	1.08	2.78
miR-885-5p	uccauuacacuacccugccucu	0.99	−2.94
miR-451a	aaaccguuaccauuacugaguu	0.94	−1.61
miR-222-3p	agcuacaucuggcuacugggu	0.88	−1.54
miR-140-3p	uaccacaggguagaaccacgg	0.77	−2.04

**Table 3:** Targets of the eight miRNAs contained in the predictive model of post-surgical PE in glioma patients. Target proteins were identified in miRWalk 2.0 and were combined with the complement and coagulation cascades pathway from KEGG. Validated targets are defined as those that have been experimentally proven to be regulated by a miRNA. Predicted targets are defined as those that have been theoretically identified based on the free binding energy between the miRNA and the presumed target mRNA sequence.

	Complement and Coagulation Cascades Pathway	
miRNA	Predicted Target	Validated Target
miR-363-3p	C4BPA, CR2, CD55, KNG1	-
miR-93-3p	PROS1, TFPI, MASP1, F9, C6, C8B, MBL2	-
miR-22-5p	TFPI, F11, C8B	-
miR-130b-3p	SERPINA1, SERPING1, C3, MBL2, SERPINE1, C8A	F3
miR-885-5p	CD59, CFI, KNG1	-
miR-451a	-	-
miR-222-3p	-	-
miR-140-3p	CD59, SERPINA1, MASP1	-

**Table 4:** miRNAs included in the Random Forest regression predictive model of post-surgical PE in meningioma patients attained in the screening stage. miRNA sequences detailed in accordance with miRBase 22.1. Fold-change expresses the ratio of the average expression level of a miRNA in meningioma patients who suffered a post-surgical PE event and those who did not.

miRNA	Sequence	Fold-Change
miR-29a-3p	uagcaccaucugaaaucgguua	1.57
miR-660-5p	uacccauugcauaucggaguug	−1.59
miR-331-3p	gccccugggccuauccuagaa	2.20
miR-126-5p	cauuauuacuuuugguacgcg	1.99
miR-23a-3p	aucacauugccagggauuucc	1.91
miR-23b-3p	aucacauugccagggauuaccac	1.95

**Table 5:** Targets of the six miRNAs contained in the predictive model of post-surgical PE in meningioma patients before surgery. Target proteins were identified in miRWalk 2.0 and were combined with the complement and coagulation cascades pathway from KEGG. Validated targets are defined as those that have been experimentally proven to be regulated by a miRNA. Predicted targets are defined as those that have been theoretically identified based on the free binding energy between the miRNA and the presumed target mRNA sequence.

	Complement and Coagulation Cascades Pathway	
miRNA	Predicted Target	Validated Target
miR-29a-3p	BDKRB1, CR1, KNG1, BDKRB2, C8G	FGA, FGB, FGG
miR-660-5p	C9, KNG1	-
miR-331-3p	C3AR1, CFB, F10, F11, KLKB1, SERPINF2, F7	-
miR-126-5p	CR2, F8, F9	-
miR-23a-3p	CR1, F11, F2R, F8, MBL2, PLAU, PLAUR, PROS1, C1S, SERPINC1	-
miR-23b-3p	CR1, F11, F2R, F8, MBL2, PLAUR, PROS1, C1S, SERPINC1	PLAU

## Supplementary Materials

The following are available online at https://www.mdpi.com/2072-6694/12/6/1536/s1, Figure S1: Differences in the expression level of three synthetic spike-in RNAs among glioma and meningioma patients who suffered post-surgical PE and those who did not. (A) Spike-in 2 monitors the RNA isolation step. (B) Spike-in 6 monitors the retrotranscription efficiency. (C) Spike-in 3 functions as inter-plate calibrator to evaluate qPCR performance. Expression levels are represented as Ct values. Figure S2: Selection of candidate miRNA normalizers and analysis of their stability conducted with the comprehensive tool RefFinder. (A) Stability value of the 5 candidate miRNAs normalizers proposed, rendered by the Comprehensive Ranking of RefFinder. The lower the stability value, the higher the stability of each miRNA. (B) Ct values of the 5 candidate miRNAs normalizers proposed arranged from the most stable miRNA (miR-93-5p) to the less stable miRNA (miR-103-3p). The lower the Ct value, the higher the expression level of a miRNA. Figure S3: Sensitivity and Specificity Profile Plot of the multivariable elastic net logistic regression predictive model that includes 6 miRNAs measured before surgery (miR-363-3p, miR-93-3p, miR-22-5p, miR-451a, miR-222-3p and miR-140-3p) as predictors of post-surgical PE in glioma patients. Figure S4: Predictive model obtained with the Random Forest regression with miRNAs measured before surgery as predictors of post-surgical PE in meningioma patients. Figure S5: ROC curve of the Khorana score as predictor of post-surgical PE in glioma patients. Table S1: Database including the clinical variables and markers studied in glioma and meningioma patients. Table S2: Differences in the baseline clinical characteristics in glioma patients according to the occurrence of PE. Table S3: Differences in the baseline clinical characteristics in meningioma patients according to the occurrence of PE.
