# Supplementary material for: microRNAs and Markers of Neutrophil Activation as Predictors of Early Incidental Post-Surgical Pulmonary Embolism in Patients with Intracranial Tumors
Source: Cancers (Basel). 2020 Jun 11;12(6):1536. doi: 10.3390/cancers12061536 (PMC7353032; doi:10.3390/cancers12061536)
Supplement: Supplementary file 1 [file cancers-12-01536-s001.zip › Supplementary figures & Tables final.docx]

Supplementary Materials

microRNAs and Markers of Neutrophil Activation as Predictors of Early Incidental Post-Surgical Pulmonary Embolism in Patients with Intracranial Tumors


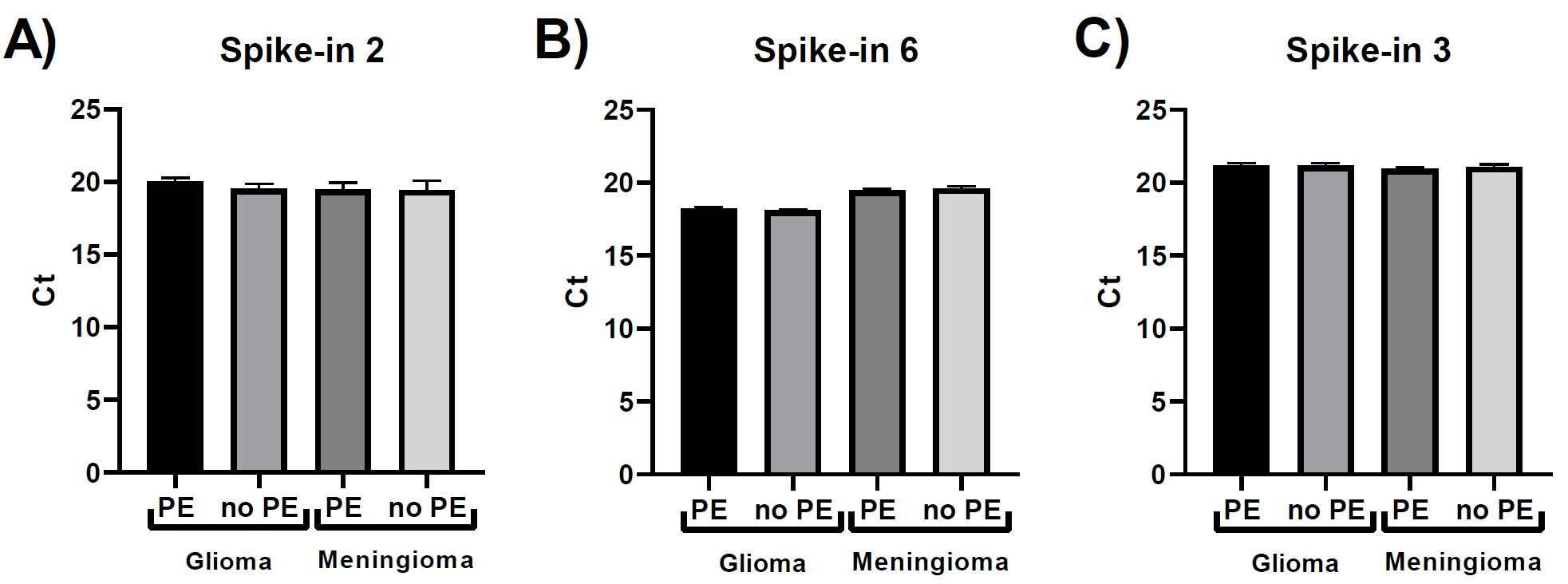


**Figure S1.** Differences in the expression level of three synthetic spike-in RNAs among glioma and meningioma patients who suffered post-surgical PE and those who did not. A) Spike-in 2 monitors the RNA isolation step. B) Spike-in 6 monitors the retrotranscription efficiency. C) Spike-in 3 functions as inter-plate calibrator to evaluate qPCR performance. Expression levels are represented as Ct values.


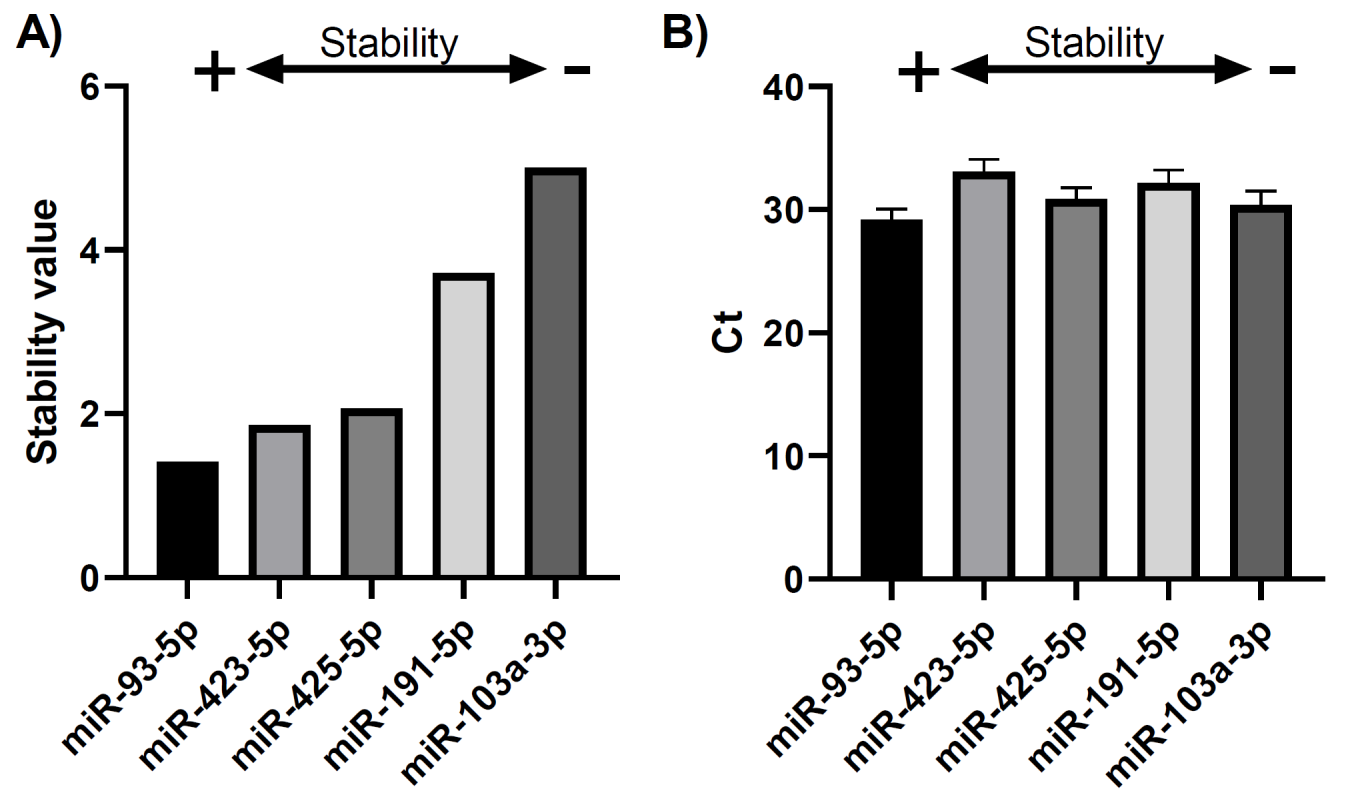


**Figure S2.** Selection of candidate miRNA normalizers and analysis of their stability conducted with the comprehensive tool RefFinder. A) Stability value of the 5 candidate miRNAs normalizers proposed, rendered by the Comprehensive Ranking of RefFinder. The lower the stability value, the higher the stability of each miRNA. B) Ct values of the 5 candidate miRNAs normalizers proposed arranged from the most stable miRNA (miR-93-5p) to the less stable miRNA (miR-103-3p). The lower the Ct value, the higher the expression level of a miRNA.


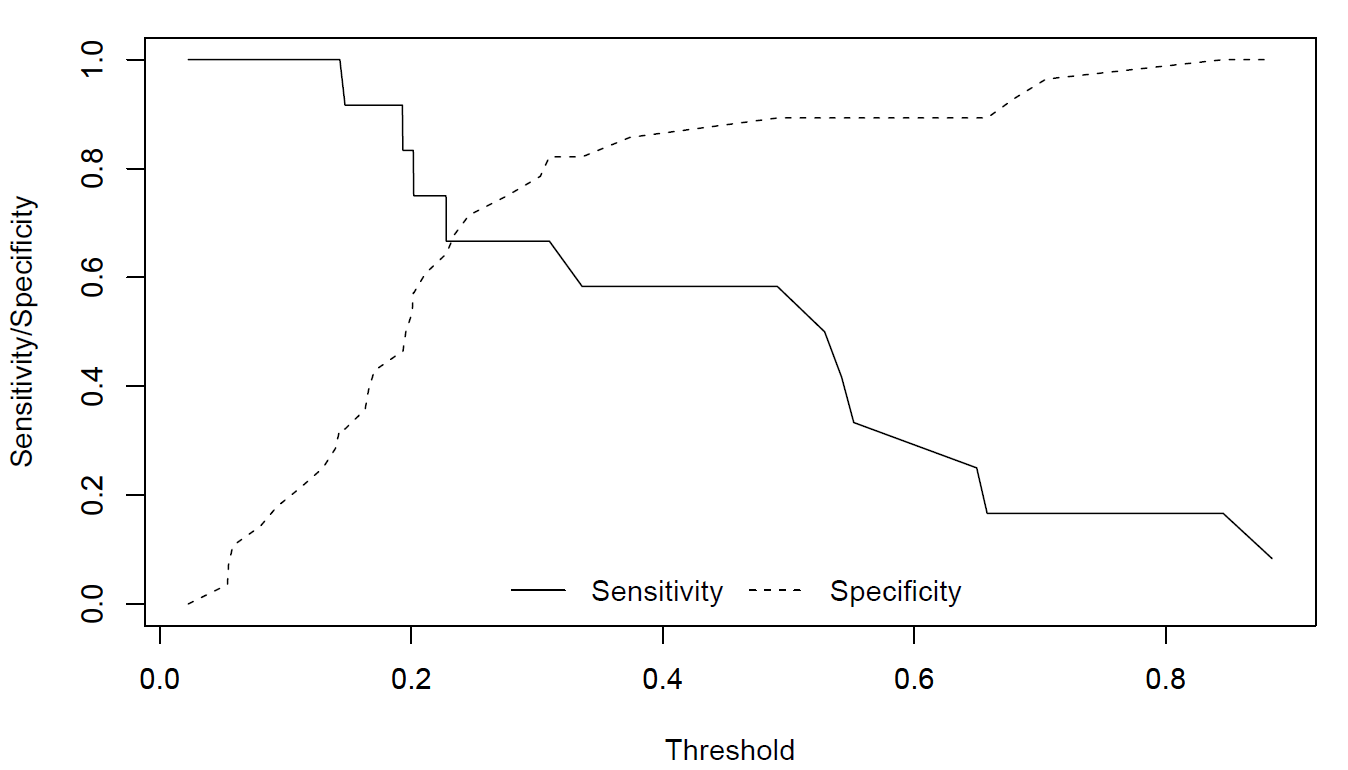


**Figure S3.** Sensitivity and Specificity Profile Plot of the multivariable elastic net logistic regression predictive model that includes 6 miRNAs measured before surgery (miR-363-3p, miR-93-3p, miR-22-5p, miR-451a, miR-222-3p and miR-140-3p) as predictors of post-surgical PE in glioma patients.


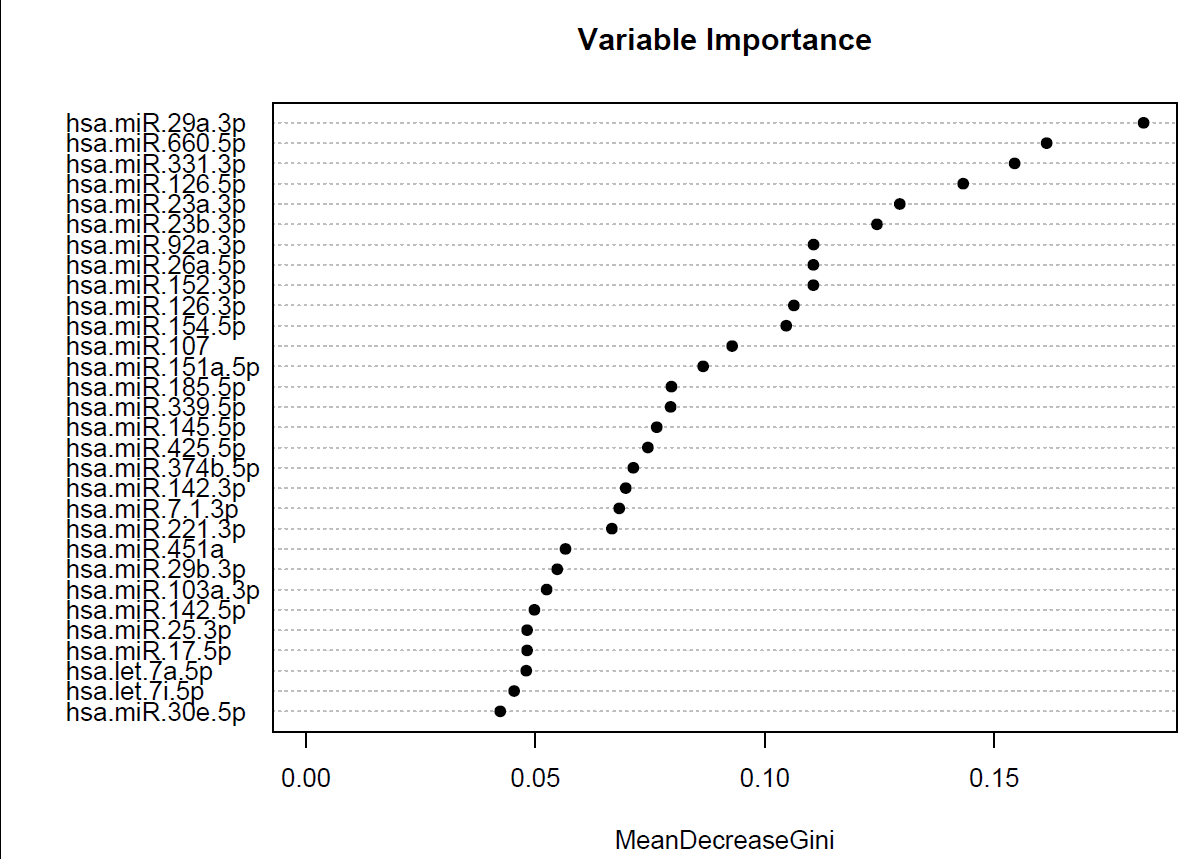


**Figure S4.** Predictive model obtained with the Random Forest regression with miRNAs measured before surgery as predictors of post-surgical PE in meningioma patients.


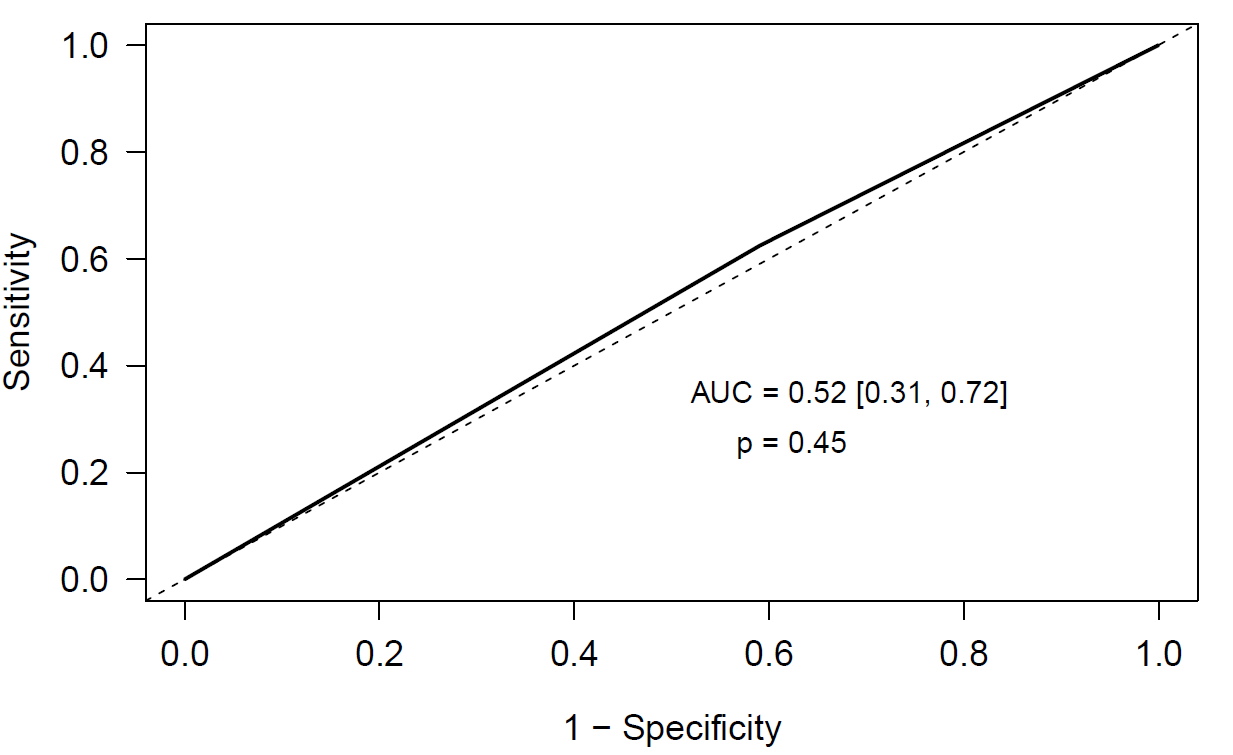


**Figure S5.** ROC curve of the Khorana score as predictor of post-surgical PE in glioma patients.

**Table S2*.*** Differences in the baseline clinical characteristics in glioma patients according to the occurrence of PE.

| **Clinical Characteristic** | **Glioma Patients with PE**  **(N = 17)** | **Glioma Patients without PE**  **(N = 33)** | **P-value ^¥^** |
| --- | --- | --- | --- |
| Age, y | 65 (56–71) | 56 (49–69) | 0.2473 |
| Female sex, N (%) | 7 (41) | 15 (45) | >0.9999 |
| BMI, Kg/m^2^ | 24.9 (23.9–27.6) | 23.9 (21.6–27.1) | 0.2226 |
| Comorbidities, N (%)  Cardiovascular  Respiratory  Metabolic *  Miscellanea ^†^ | 9 (53)  0 (0)  1 (6)  3 (18) | 11 (33)  1 (3)  7 (21)  2 (6) | 0.2293  >0.9999  0.2374  0.3215 |
| Pre-operative KPS ≥ 80, N (%) | 17 (100) | 30 (91) | 0.1998 |
| Post-operative KPS ≥ 80, N (%) | 15 (88) | 29 (88) | 0.9707 |
| WHO classification, N (%)  Grade I  Grade II  Grade III  Grade IV | 0 (0)  0 (0)  1 (6)  16 (94) | 0 (0)  6 (18)  7 (21)  20 (61) | >0.9999  0.0833  0.2374  0.0183 |
| Tumor location, N (%)  Skull base  Cerebral convexity-falx  Superficial  Deep-seated | 0 (0)  0 (0)  4 (24)  13 (76) | 0 (0)  0 (0)  8 (24)  25 (76) | >0.9999  >0.9999  >0.9999  >0.9999 |
| Tumor dimension, cm^3^ | 24.8 (15.5–64.1) | 24.1 (10.0–49.3) | 0.4518 |
| Duration of surgery, min | 228 (199–240) | 240 (203–258) | 0.5828 |
| Khorana score, N (%)  0  1 | 6 (46)  7 (54) | 16 (59)  11 (41) | 0.5092 |
| Hemoglobin, g/dL | 14.1 (12.5–15.1) | 13.6 (12.7–14.8) | 0.6480 |
| WBC, × 10^3^/mmc | 10.9 (8.8–12.7) | 9.3 (6.4–12.0) | 0.1283 |
| Neutrophils, × 10^3^/mmc | 6.52 (5.26–7.63) | 5.60 (3.85–7.18) | 0.1345 |
| Platelets, × 10^3^/mmc | 225.0 (206.0–252.5) | 227.0 (179.5–247.0) | 0.5366 |
| PT ratio | 0.97 (0.92–1.10) | 0.99 (0.93–1.09) | 0.5271 |
| APTT ratio | 0.77 (0.69–0.82) | 0.84 (0.77–0.93) | 0.0139 |
| Fibrinogen, mg/dL | 229 (197–271) | 223 (186–288) | 0.7419 |
| D-dimer, ng/mL | 295 (128–725) | 193 (93–537) | 0.2895 |
| CRP, mg/L | 0.06 (0.03–0.24) | 0.07 (0.03–0.19) | 0.6419 |
| eGFR, mL/min/1.73 m^3^ | 86.5 (74.8–107.0) | 99.8 (81.2–121.1) | 0.2644 |

Continuous variables are displayed as median and interquartile range. Categorical variables are displayed as count and percentage. ^¥^ P-values for continuous variables were calculated with the Kruskal-Wallis test and P-values for categorical variables were calculated with the Fisher´s exact test. * Diabetes mellitus, hypercholesterolemia, obesity, hyper- or hypothyroidism, chronic liver or renal disease. **^†^** Previous surgeries, other neoplasms, psychiatric disorders. PE, pulmonary embolism; BMI, body mass index; KPS, Karnofsky Performance Status; WHO, World Health Organization Classification of brain tumors; WBC, white blood cells; PT, prothrombin time; APTT, activated partial thromboplastin time; CRP, C-reactive protein; eGFR, estimated glomerular filtration rate.

**Table S3.** Differences in the baseline clinical characteristics in meningioma patients according to the occurrence of PE.

| **Clinical characteristic** | **Meningioma Patients with PE**  **(N = 17)** | **Meningioma Patients without PE**  **(N = 33)** | **P-value ^¥^** |
| --- | --- | --- | --- |
| Age, y | 54 (49–66) | 66 (52–72) | 0.1588 |
| Female sex, N (%) | 12 (71) | 21 (64) | 0.7568 |
| BMI, Kg/m^2^ | 24.2 (21.1–29.4) | 26.2 (21.8–29.7) | 0.3983 |
| Comorbidities, N (%)  Cardiovascular  Respiratory  Metabolic *  Miscellanea ^†^ | 4 (24)  0 (0)  1 (6)  4 (24) | 14 (42)  2 (6)  4 (12)  1 (3) | 0.2272  0.5420  0.5625  0.0400 |
| Pre-operative KPS ≥ 80, N (%) | 17 (100) | 32 (97) | 0.4684 |
| Post-operative KPS ≥ 80, N (%) | 15 (88) | 32 (97) | 0.2180 |
| WHO classification, N (%)  Grade I  Grade II  Grade III  Grade IV | 15 (88)  2 (12)  0 (0)  0 (0) | 30 (91)  3 (9)  0 (0)  0 (0) | >0.9999  >0.9999  >0.9999  >0.9999 |
| Tumor location, N (%)  Skull base  Cerebral convexity-falx  Superficial  Deep-seated | 2 (12)  15 (88)  0 (0)  0 (0) | 13 (39)  20 (61)  0 (0)  0 (0) | 0.0555  0.0555  >0.9999  >0.9999 |
| Tumor dimension, cm^3^ | 22.3 (6.8–40.2) | 9.7 (5.0–29.2) | 0.1533 |
| Duration of surgery, min | 280 (188–473) | 205 (163–250) | 0.00258 |
| Khorana score, N (%)  0  1 | 10 (77)  3 (23) | 26 (84)  5 (16) | 0.6760 |
| Hemoglobin, g/dL | 13.0 (11.8–14.2) | 12.9 (12.0–13.9) | 0.7053 |
| WBC, × 10^3^/mmc | 6.0 (5.0–9.6) | 6.1 (4.7–7.3) | 0.4851 |
| Neutrophils, × 10^3^/mmc | 3.64 (3.01–5.95) | 3.66 (2.84–4.41) | 0.4203 |
| Platelets, × 10^3^/mmc | 263.0 (214.5–306.8) | 208.5 (167.5–234.8) | 0.0029 |
| PT ratio | 1.01 (0.99–1.06) | 1.04 (0.97–1.09) | 0.5968 |
| APTT ratio | 0.95 (0.86–1.03) | 0.94 (0.85–1.01) | 0.8262 |
| Fibrinogen, mg/dL | 257 (239–296) | 257 (214–310) | 0.6877 |
| D-dimer, ng/mL | 183 (108–243) | 165 (88–206) | 0.6063 |
| CRP, mg/L | 0.13 (0.06–0.29) | 0.09 (0.06–0.24) | 0.5331 |
| eGFR, mL/min/1.73 m^3^ | 91.9 (76.3–113.9) | 90.9 (78.3–97.8) | 0.8136 |

Continuous variables are displayed as median and interquartile range. Categorical variables are displayed as count and percentage. ^¥^ P-values for continuous variables were calculated with the Kruskal-Wallis test and P-values for categorical variables were calculated with the Fisher´s exact test. * Diabetes mellitus, hypercholesterolemia, obesity, hyper- or hypothyroidism, chronic liver or renal disease. **^†^** Previous surgeries, other neoplasms, psychiatric disorders. PE, pulmonary embolism; BMI, body mass index; KPS, Karnofsky Performance Status; WHO, World Health Organization Classification of brain tumors; WBC, white blood cells; PT, prothrombin time; APTT, activated partial thromboplastin time; CRP, C-reactive protein; eGFR, estimated glomerular filtration rate.
